# Individualized cortico-basal ganglia network effective connectivity predicts outcomes of STN-DBS in patients with Parkinson's disease

**DOI:** 10.3389/fnins.2025.1745334

**Published:** 2026-01-14

**Authors:** Yu Diao, Weihao Liu, Tianqi Hu, Houyou Fan, Bifa Fan, Jianguo Zhang

**Affiliations:** 1 Department of Pain Management, China-Japan Friendship Hospital, Beijing, China; 2 Department of Neurosurgery, Beijing Friendship Hospital, Capital Medical University, Beijing, China; 3 Department of Functional Neurosurgery, Beijing Tiantan Hospital, Capital Medical University, Beijing, China; 4 Department of Functional Neurosurgery, Beijing Neurosurgical Institute, Capital Medical University, Beijing, China

**Keywords:** cortico-basal ganglia network, deep brain stimulation, individual, Parkinson's disease, volume of tissue activated

## Abstract

**Background:**

Deep brain stimulation (DBS) of the subthalamic nucleus (STN) is an effective treatment for Parkinson's disease (PD) patients. However, postoperative outcomes vary with no reliable predictive method.

**Methods:**

Our study involves 43 PD patients undergoing STN-DBS. Preoperative resting-state functional magnetic resonance imagings (rs-fMRI) were collected. The volume of tissue activated (VTA) was defined based on contact points and stimulation parameters. A model of the cortico-basal ganglia network was established using dynamic causal modeling. The correlation between the UPDRS-III and the network edges was determined through Pearson correlation analysis. Furthermore, a generalized linear model was employed to predict the post-DBS motor improvement.

**Results:**

Individual STN-VTA intersections were found to be important to UPDRS-III improvement induced by DBS (R = 0.59, *P* = 0.001). STN-VTA intersections were related to the thalamic-primary motor cortex (M1) (R = 0.47, *P* = 0.005), and M1-STN (R = 0.40, *P* = 0.006) coupling strength. The coupling strength of Thal-M1 (R = 0.442, *P* = 0.009) and M1-STN (R = 0.481 *P* = 0.004) resulted in DBS-induced movement enhancement, particularly rigidity. The strength of effective connections within the STN-Thal-M1 pathway was found to predict improvements in UPDRS-III scores (*P* = 0.003).

**Conclusion:**

Our study confirmed the relationship between clinical improvements in STN-DBS and target location as well as the stimulation parameters. By constructing personalized cortical-basal ganglia network models based on target location as well as the stimulation parameters, we discovered that the effective connection strength in STN-THA-M1 can predict motor improvement in PD patients undergoing STN-DBS.

## 1 Introduction

Deep brain stimulation (DBS) of the subthalamic nucleus (STN) has proven to be a highly effective treatment for addressing motor impairments in patients with Parkinson's disease (PD). Nevertheless, the postoperative effects vary considerably among patients (Diao et al., 2024a). While levodopa responsiveness is a common assessment method employed to predict DBS effectiveness, significant differences in treatment responses persist among patients following STN-DBS (Li et al., 2022).

It is widely assumed that the success of DBS is heavily reliant on stimulation in the best location inside the target region. Clinical studies reported that when the electrode position was located in the dorsolateral motor-connected segment of the STN, the patients' motor disorders improved the most (Mathiopoulou et al., 2022; Wei et al., 2021). Furthermore, more anterior and ventral stimulation of STN improves the patients' quality of life (Jost et al., 2024). Stimulation of the ventral border region of the STN and the sensorimotor STN has also been linked to improvements of mood and apathy (Petry-Schmelzer et al., 2019).

In addition to location, parameter settings also have an effect on postoperative efficacy, and patients can achieve optimal motor improvement only when optimal parameter settings are used. To better identify the region stimulated by DBS, the concept of the volume of tissue activated (VTA) was introduced. Electrode placement, contact selection, and parameter settings together determine the range and location of the stimulus, which can be visualized using the VTA (Roediger et al., 2022; Waldthaler et al., 2021; Lai et al., 2021). Stimulating different positions of the STN can cause differences in clinical symptoms, which are based on structural connections. The fibrous bundles in and around the nucleus travel through the VTA, form fiber bundles with different cortical structures, and ultimately result in different postoperative outcomes (Lai et al., 2021; Vassal et al., 2020; Strotzer et al., 2022). For example, by stimulating fibrous tracts structurally connected to the M1, STN-DBS reduces the MDS Unified Parkinson's Disease Rating Scale (MDS-UPDRS)-III (Vassal et al., 2020) number. However, when VTA covers the internal capsule fiber, it can cause paresthesia or dysarthria (Strotzer et al., 2022; Candeias da Silva et al., 2025; Diao et al., 2024b).

STN-DBS has been demonstrated to induce distal responses in multiple brain regions in PD patients, including in the thalamus, striatum, supplementary motor area (SMA), temporal cortex, primary motor cortex (M1), and others (Yu et al., 2022; Chen et al., 2022), suggesting the presence of extensive functional connectivity networks connecting the STN with the cortex. Furthermore, the effectiveness of improving motor symptoms is closely linked to the degree of functional and structural connectivity between the STN and different brain regions, such as M1 and SMA (Chen et al., 2022; Horn et al., 2019). To the best of our knowledge, such studies were typically based on individualized positioning and database functional connectivity maps, so predictive analysis based on individual functional connectivity data was still lacking (Xu et al., 2018). On this basis, a study recently attempted to predict the efficacy of DBS at the individual level, using the functional connection between STN and GPi level (Younce et al., 2021). However, it is unknown whether the cortico-basal ganglia individualized functional network of ganglia can predict motor efficacy.

In our study, we therefore used 43 individual PD resting-state functional magnetic resonance imaging (rs-fMRI) data to construct a cortico-basal ganglia network, and investigate relationship between the individual network connectivity and clinical benefits. We hypothesized that specific individualized connectivity features of the cortico-basal ganglia network could predict postoperative outcomes.

## 2 Method

### 2.1 Participants

A cohort of 43 patients (comprising 20 males and 23 females) underwent bilateral DBS implantation surgery at Beijing Tiantan Hospital for PD. These patients voluntarily participated in our study. The research protocol for each patient consisted of two key components: (1) preoperative rs-fMRI and (2) clinical assessments of the effects of STN-DBS. To ensure consistent conditions, all medication was withdrawn overnight prior to the scanning. Inclusion criteria for patients involved their ability to recline comfortably with minimal head tremors while off medication and under stimulation. Ethical approval for this study was obtained from the Beijing Tiantan Hospital, and all patients provided written informed consent, adhering to the principles of the Declaration of Helsinki.

### 2.2 Clinical assessment

The assessment of motor functions was carried out using the Movement Disorder Society Unified Parkinson's Disease Rating Scale part III (MDS-UPDRS-III). This scale comprises three distinct domains (Hariz et al., 2021), which include rigidity (item 3), tremor (items 15–18) and bradykinesia (items 2, 4–8, and 14). Preoperative assessment was conducted before surgery. Clinicians programmed the optimal parameters approximately 4 weeks after surgery. A follow-up assessment was performed within 8–12 months of the initial stimulation.

### 2.3 MRI and computed tomography acquisitions

Patients underwent scanning using a 3.0-T Prisma MRI scanner (Siemens, The Hague, The Netherlands). We obtained the rs-fMRI data 1–2 weeks before neurosurgery. During rs-fMRI, patients were instructed to recline in the scanner with their eyes open and were asked to remain awake. Structural images were obtained using the magnetization-prepared rapid acquisition gradient echo (MPRAGE) sequence with the following parameters: a repetition time (TR) of 1.56 ms, an echo time (TE) of 1.69 ms, a flip angle (FA) of 8°, a field of view (FOV) measuring 100 mm × 100 mm, and a matrix size of 256 mm × 256 mm. On the other hand, blood-oxygen-level-dependent (BOLD) images were acquired using an echo-planar imaging (EPI) sequence with the following parameters: a TR of 750 ms, a TE of 30 ms, a flip angle of 54°, an acquisition matrix of 74 × 74, an FOV of 100 mm × 100 mm, 16 axial slices with a thickness of 3 mm, and a scanning time approximately equal to 360 s. A thin slice (0.625 mm) CT image was further obtained using General Electric Company (GE) equipment 1 month after the DBS procedure.

### 2.4 Individualization of the VTA

**Figure 1:**
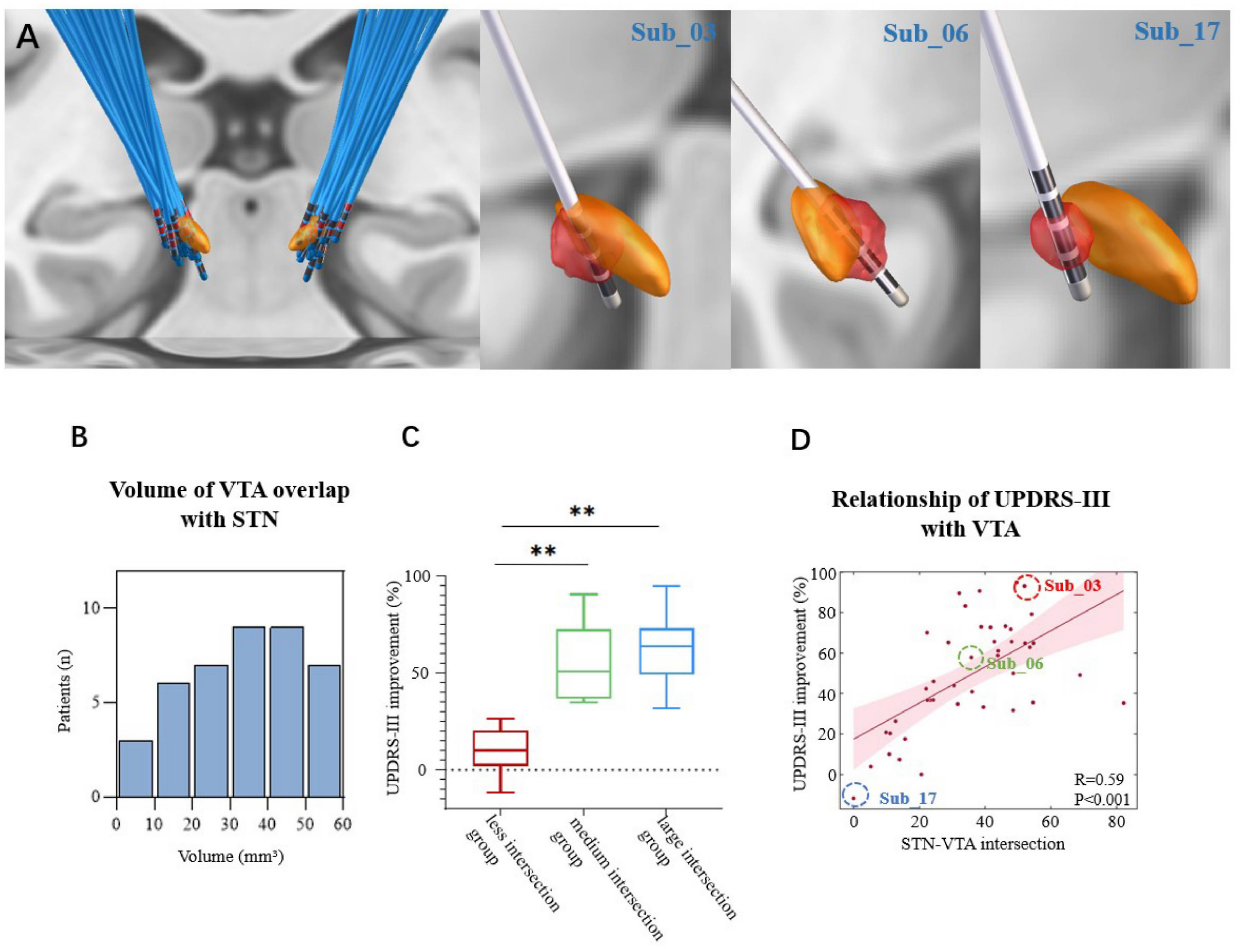
**(A)** STN lead localization in all subjects, and orange structures are bilateral STN **(Left)**. Volumes of Tissue Activation (VTA) (red structure) in Sub_03 which overlap with the STN about 49.68 mm^3^. VTA in Sub_06 which overlap with the STN about 35.84 mm^3^; and VTA in Sub_17 showed no overlap with the STN. **(Right) (B)** Distribution of the number of patients in different STN-VTA intersections. **(C)** When the overlapping volume with the STN is less than 20 mm^3^ (less intersection group), the improvement of UPDRS-III was less than 30% and was significantly lower than that of patients with overlapping volumes of medium intersection group (20–40 mm^3^) and large intersection group (40–60 mm^3^). **P < 0.001 with one-way anowa. **(D)** The amount of stimulated STN explains improvement of UPDRS-III across patients. The larger the weighted overlap between the VTA and STN, the better the improvement in UPDRS-III.

The Lead-DBS software, available at (http://www.lead-dbs.org), was employed for electrode localization in both cohorts (Horn et al., 2019). This process encompasses several essential steps, including registration, correction for brain shift, normalization, electrode positioning, result verification, manual adjustments, and more, all of which collectively contribute to the post-DBS electrode reconstruction (Sobesky et al., 2022). Figure 1A provides a visual representation of the electrode implantation procedure for all patients. In each patient's case, the volume of tissue activated (VTA) was computed using a finite element method (FEM)-based model (Horn et al., 2019).

Subsequently, according to the overlapping volumes of the VTA and STN, we divided them into less intersection (below 20 mm^3^), medium intersection (20–40 mm^3^), and large intersection groups (40–60 mm^3^).

### 2.5 The fMRI data preprocessing and denoising

#### 2.5.1 Basic spatial preprocessing

All functional images underwent the following initial spatial preprocessing steps using SPM12:

(a) Discarding of Initial Volumes: The first 20 volumes of each run were discarded to allow for magnetic field stabilization.

(b) Realignment: All remaining volumes were realigned to the first volume to correct for head motion. Six rigid-body motion parameters (three translations and three rotations) were estimated. Framewise displacement (FD) was calculated for each participant as a summary metric of scan-to-scan head motion. Participants exhibiting translational or rotational head motion exceeding 3 mm or 3°, respectively, in any direction were excluded from further analysis.

(c) Co-registration and Normalization: Individual high-resolution T1-weighted structural images were co-registered to the mean realigned functional image. The structural images were then segmented and normalized to the Montreal Neurological Institute (MNI) standard space using the DARTEL algorithm. The resulting nonlinear transformation fields were applied to all functional images.

(d) Spatial Smoothing: Normalized functional images were spatially smoothed using a 6-mm full-width at half-maximum (FWHM) Gaussian kernel.

#### 2.5.2 Temporal denoising and nuisance regression

Following spatial preprocessing, a rigorous multi-step temporal denoising procedure was applied to isolate neural BOLD fluctuations from non-neural artifacts (Ciric et al., 2017).

(a) Motion artifact removal via ICA-AROMA: Smoothed functional data were processed using FSL's ICA-AROM. This data-driven, single-subject independent component analysis (ICA) method automatically identifies and removes motion-related noise components without requiring a classifier trained on external data.

(b) Physiological noise regression: To account for residual physiological noise (e.g., cardiac, respiratory rhythms), average time series were extracted from eroded cerebrospinal fluid (CSF) and white matter (WM) masks derived from the T1 segmentation. These signals, along with their first derivatives, were regressed from the ICA-AROMA-cleaned data.

(c) Scrubbing: A scrubbing procedure was implemented to minimize the impact of high-motion volumes on connectivity estimates. Any volume with a FD exceeding 0.2 mm, as well as the one volume preceding and two volumes following it, were flagged as “bad”. These volumes were subsequently excluded from the calculation of correlation matrices for functional connectivity. The proportion of scrubbed volumes was recorded for each participant, and all included participants retained sufficient data (> 5 minutes of clean data) for stable estimation.

(d) Temporal Filtering: Finally, the residual time series were bandpass-filtered within a frequency range of 0.01–0.08 Hz to retain low-frequency fluctuations while removing low-frequency drift and high-frequency noise.

#### 2.5.3 Quality control

The results of all preprocessing and denoising stages, including registration accuracy, component classification by ICA-AROMA, and the final cleaned time series, were subjected to thorough visual inspection to ensure data quality. The cleaned, denoised BOLD time series were used for all subsequent seed-based functional connectivity and dynamic causal modeling (DCM) analyses.

### 2.6 VTA-based personalized functional connectivity and ROI localization

To build the cortico-basal ganglia network, regions of interest (ROIs) were first defined based on our own data. Briefly, we calculated the functional connectivity of the bilateral individualized VTA to the whole brain using REST (https://rfmri.org/REST) (Hou et al., 2016), then used the FWE correction to identify regions that were correlated with the VTA. Patients with a VTA/STN overlapping less than 20 mm^3^ were excluded in establishing a realistic STN projection model. The VTA-related regions mainly included the bilateral motor thalamus, bilateral caudate nucleus, bilateral pallidus, temporal lobe, insula, occipital lobe, SMA, primary motor cortex, and motor cerebellum. To the best of our knowledge, the major motor symptoms of PD are caused by an imbalance in the excitability of striatal efferent projection systems in the basal ganglia circuit (Tanimura et al., 2022). In addition, after dopamine depletion, the synaptic strengths from intralaminar thalamic neurons to indirect-pathway medium spiny neurons were preferentially reduced, potentially contributing to the imbalance between direct and indirect pathways (Parker et al., 2016). Therefore, the STN, putamen, and thalamus were chosen as ROIs in direct and indirect pathways. Furthermore, considering the hyperdirect pathway and motor circuit, M1 was set as a cortical ROI (Filion, 2000). The cerebellum was also included in the ROI due to its recently established important role in PD motor symptoms.

**Figure 2:**
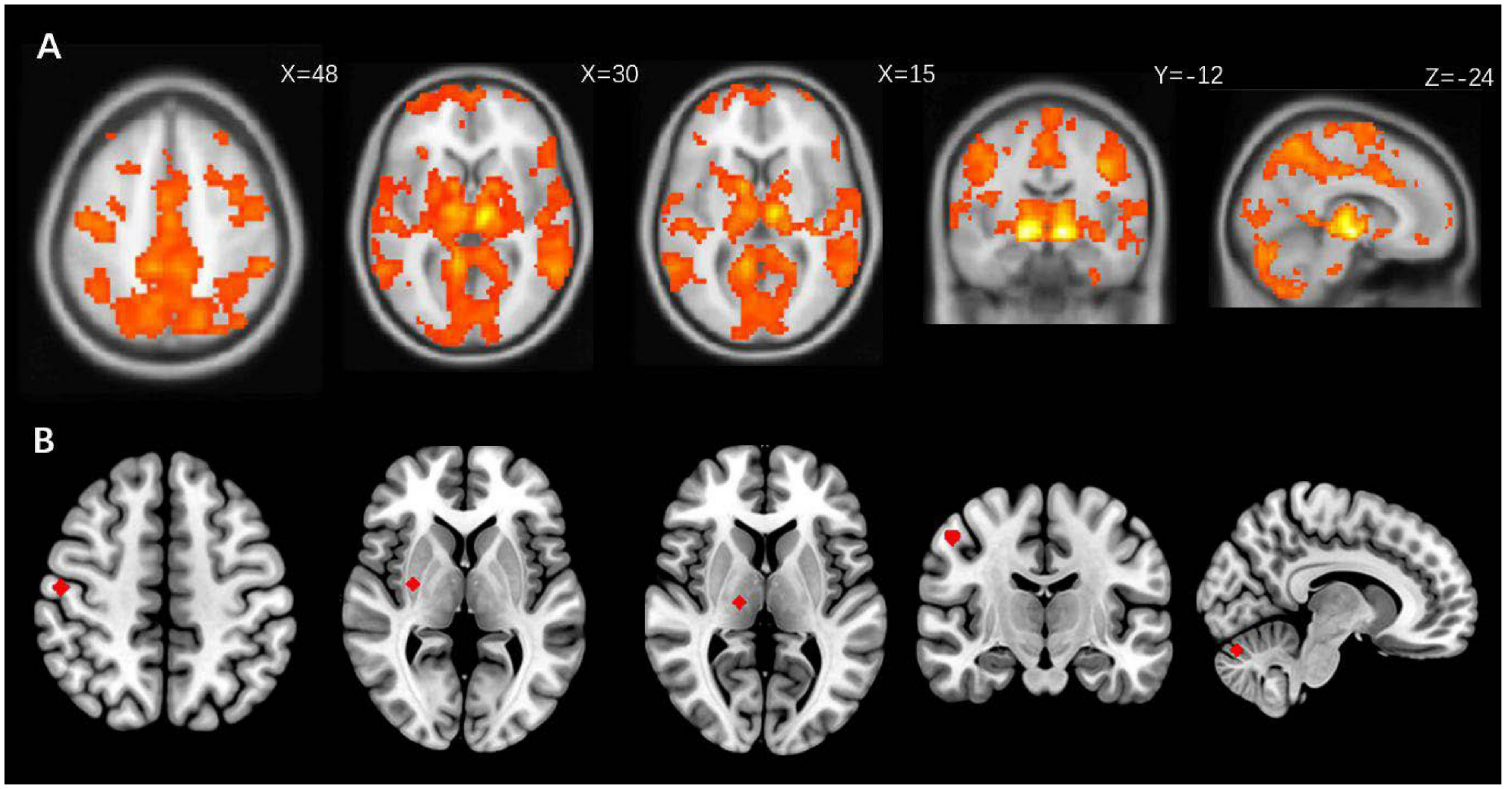
**(A)** The M1, putamen, thalamus and cerebellum are positive correlated with bilateral VTA overlapping with STN more than 20 mm^3^ in resting state connectivity, threshold at a voxel level of *P* < 0.05 (FWE). **(B)** is a schematic diagram of the ROI extraction in the right brain that is correlated with the individualized STN.

Based on the functional connectivity between the VTA and the thalamus, and the results of previous studies on the white matter fibers connecting the ventral lateral thalamus (VLP) and the M1, we were able to determine the coordinates of the thalamus. M1 was extracted, and the peak T statistic within the mask was used as the central coordinate. The cerebellum was selected using the peak T statistic on the upper part of the cerebellar cortex as the central coordinate for subsequent analysis. It is currently believed that this region is associated with improvement of axial symptoms with DBS. In addition, the putamen was extracted using the peak T statistic within the mask, resulting in four volumes of interest, each with a radius of 4 millimeters (M1, putamen, thalamus, and cerebellum) for each patient. Finally, to characterize true motor network projections of the STN at individual levels, we used VTA as the STN ROI mask. The selection of ROIs is shown in Figure 2.

### 2.7 DCM of the cortico-basal ganglia network

The DCM model can simulate efficient cortico-basal ganglia network connectivity in patients in a low frequency BOLD manner (Friston et al., 2003). The purpose was to elucidate the observed neuroimaging data by examining the coupling, or effective connectivity, within and between brain regions within a network. In the context of DCM, effective connectivity serves to quantify the causal influence that one brain region exerts on either itself or on another region in the network (Horn et al., 2019; Kahan et al., 2019).

We constructed the basal ganglia-cortical circuit based on the study by Kahan et al. (Kahan et al., 2014). In this study, SPM12 (https://www.fil.ion.ucl.ac.uk/spm/) was used to invert 32 constructed cortico-basal ganglia network models, and estimation of coupling parameters and model evidence were provided (Kahan et al., 2014). A Bayesian model-selection procedure (fixed effects assumption) was used to compare free energies of each model while considering model fit and complexity. The group winner was the model with the highest model evidence. After obtaining the winning model, it was used as the final patient's cortico-basal ganglia network.

Additionally, we leveraged the cerebellum locations derived from the functional connectivity of VTA to the entire brain to construct four DCMs. These models represented pure cortico-cerebellar coupling, subcortical-cerebellar coupling, or a combination of both. We built these DCMs upon the foundation of the DCM for the cortico-basal ganglia network. In a similar manner, we compared models and their coupling parameters.

### 2.8 Correlation and regression analysis

We used the coupling parameters of the superior model to establish correlations with long-term patient outcomes and STN-VTA intersections. Furthermore, we extracted the coupling strengths for each edge within the cortico-basal ganglia network to compute Pearson's correlation coefficient (r) with improvements in UPDRS-III scores, tremor, rigidity, and bradykinesia, as well as the STN-VTA intersections. To account for multiple comparisons, a false discovery rate (FDR)-corrected p-value threshold of 0.05.

Additionally, for significantly correlated connections, we established a Multiple Linear Regression Model to predict improvements in UPDRS-III scores. We employed leave-one-out cross-validation to assess the model's performance. The correlation coefficient (r) and *p*-value between predicted and actual values were used for model performance evaluation.

### 2.9 Statistical analysis

We analyzed clinical benefits using SPSS statistical software for Windows, version 25 (SPSS, Chicago, IL, USA). The Shapiro–Wilk test was used to determine the normality of distribution of clinical scale results for all patients. Wilcoxon signed-rank tests or paired samples *t*-tests were used to assess changes in outcomes from baseline to follow-up. One-way analysis of variance was used to compare the volumes of three different VTA/STN overlapping group VTAs. Unless otherwise stated, the threshold was *P* < 0.05. Every statistical test was two-tailed.

## 3 Results

### 3.1 Clinical effect of STN-DBS

Table 1 outlines clinical characteristics and scores before and after DBS for the imaging cohort. Significantly, UPDRS-III, rigidity, tremor, and bradykinesia all improved compared to the baseline (*P* < 0.001). UPDRS-III showed a greater than 30% improvement in 34 out of the 43 patients, while nine patients had less than 30% improvement (Supplementary Table 1).

**Table 1:** Clinical improvements following STN-DBS.

**Items**	**Baseline**	**Follow-up**	**Improvement (%)**	***P***
LEDD	760.47 ± 329.24	745.61 ± 316.18	-	0.054
MDS-UPDRS-III (med off)	50.63 ± 19.91	24.30 ± 14.09	48.98 ± 26.85	< 0.001
Rigidity	8.30 ± 4.03	3.21 ± 2.92	41.59 ± 82.09	< 0.001
Tremor	8.93 ± 7.81	3.79 ± 4.81	47.87 ± 89.83	< 0.001
Bradykinesia	20.47 ± 9.47	9.86 ± 7.14	48.41 ± 39.14	< 0.001

Relevant values are reported as mean ± standard deviation. LEDD, levodopa equivalent daily dose; MDS-UPDRS-III, Movement Disorders Society-Unified Parkinson's Disease Rating Scale. Wilcoxon signed-rank test, respectively paired *t*-test, when parametric tests were applicable with raw *P* values. Significant correlations at *P* < 0.05. The *P* value is the statistical value of the difference between baseline and follow-up.

### 3.2 Differences in clinical efficacies based on the VTA

VTAs were constructed in accordance with the most recent programming (Supplementary Table 2) and are represented by the volume distribution (Figure 1B). The STN volume was approximately 120 mm^3^ with the DISTAL atlas using the MNI template space. We roughly divided the STN-VTA intersection volumes into three groups based on previously published reports about sweet spots (Nguyen et al., 2019; Jaradat et al., 2023): a less intersection group (below 20 mm^3^), medium intersection group (20–40 mm^3^), and large intersection group (40–60 mm^3^). Across patients, the UPDRS-III improvement of the medium and large intersection groups was significantly greater than that of the less intersection group (Figure 1C).

Figure 1 depicts these findings across groups, and highlights three examples. The overlap between the VTA and STN in the three patients (sub_03, sub_06, and sub_17; Figure 1A) belonged to the less intersection, medium intersection, and large intersection groups, respectively. They represented the three groups with placed DBS electrodes. UPDRS-III was correlated with the STN-VTA intersection in all patients (R = 0.57, *P* < 0.001; Figure 1D). There was no correlation between the UPDRS-III and STN-VTA intersection in patients with overlap volumes less than 20 mm^3^ (R = 0.15, *P* = 0.347).

Because the vast majority of VTA projections with less overlap were not based on STN, we removed the less group (*n* = 9) from subsequent statistics to better illustrate the predictive effect of the STN functional network.

### 3.3 Cortico-basal ganglia network model selection

**Figure 3:**
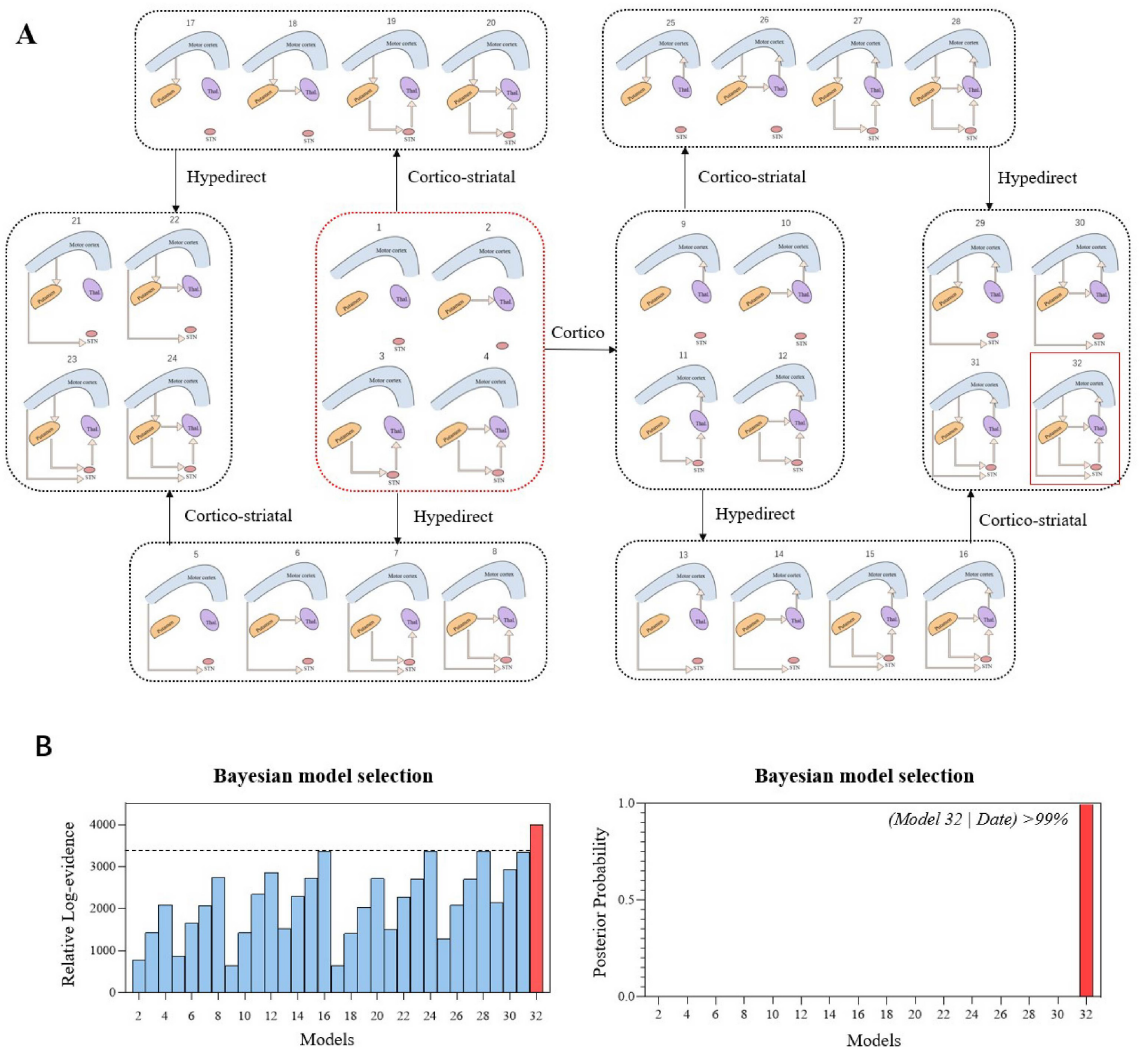
**(A)** Hypothesis space for competing models: The anatomical model of the motor cortico-basal ganglia network was simplified into 32 DCMs. These models encompass the direct pathway, indirect pathway, hyperdirect pathway, thalamus-cortical pathway, and cortico-striatal pathway. These five pathways were categorized based on the presence or absence of directional interactions in the context of PD pathology. Bayesian model selection strongly favored Model 32. **(B)** Among all the models considered, the relative log-evidence showed that Model 32 was the top-performing model, with a posterior probability of more than 99%. Model 32 displayed comprehensive connections across all five pathways of interest.

We built 32 models based on fundamental assumptions about the existence of direct and indirect pathways. The forward connections from M1 to the putamen symbolized inputs from the motor cortex into the network. We characterized the putamen with two forward connections: one linking it to the thalamus, forming what we refer to as the “direct pathway,” and another linking it to VTA, the initial component of what we term the “indirect pathway.” We also incorporated additional connections in the VTA to the thalamus, creating a second connection in our modeled indirect pathway. Furthermore, we introduced direct connections between M1 and STN to represent what we call hyperdirect pathways. In the analysis of the 32 cortico-basal ganglia network models, both fixed effects and random effects Bayesian model selection indicated that Model 32 had the highest relative log-evidence. This signifies that the likelihood of Model 32 generating the data within the model space exceeded 99% when compared to its closest competitor (Figure 3). Model 32 is characterized as a fully connected model centered on the direct and indirect pathways, establishing a greater than 99% probability of the existence of each PD patient's direct, indirect, thalamo-cortical, cortico-striatal, and hyperdirect pathways (Figure 3B).

**Figure 4:**
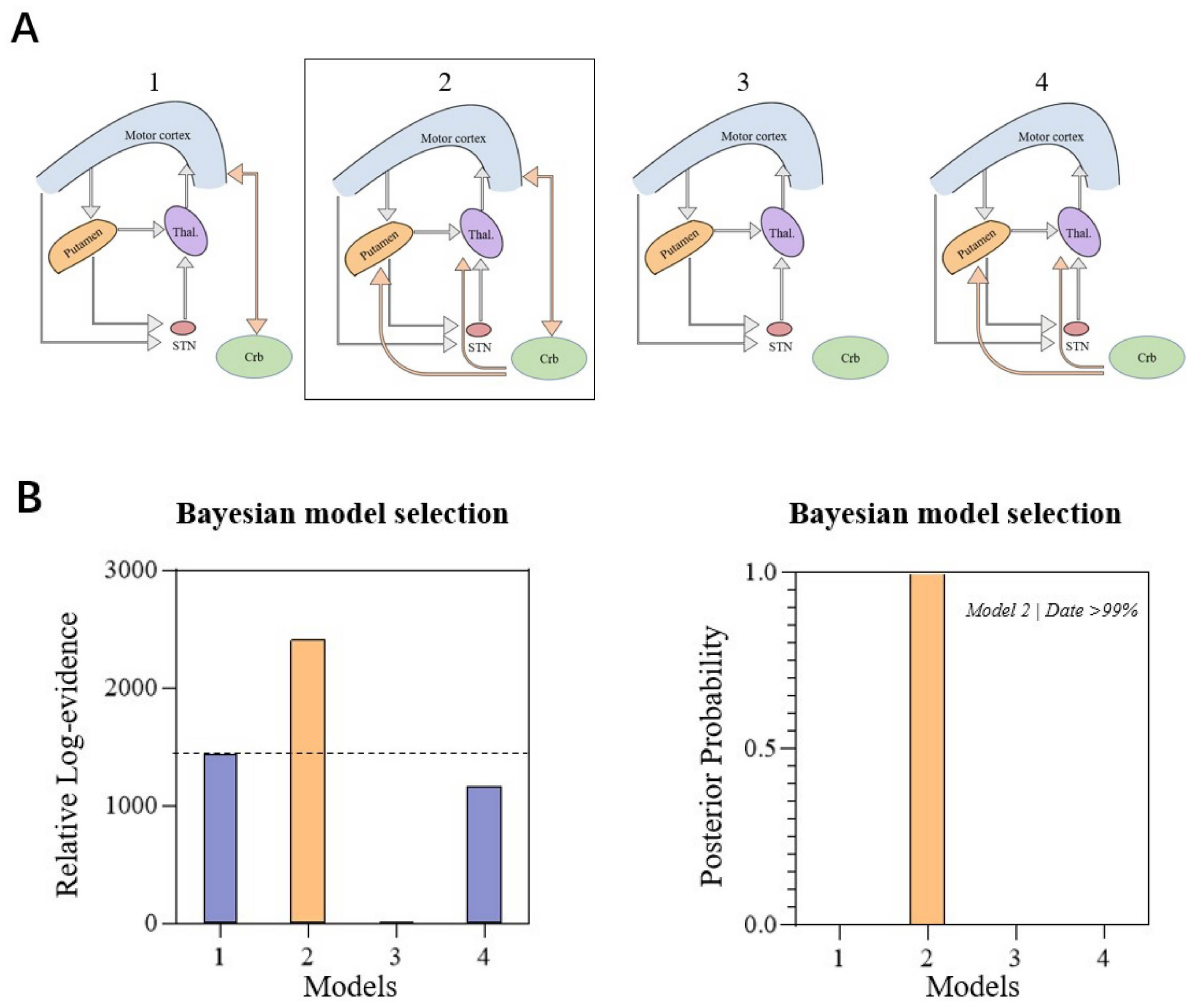
**(A)** Hypothesis space for competing models: cerebellum integrated into the cortico-basal ganglia network and considered four candidate DCM models that illustrate modulatory differences. These models encompass different types of cerebellar connectivity, including purely cortico-cerebellar coupling, subcortico-cerebellar coupling, both, or none. Bayesian model selection favored Model 2. **(B)** The relative log-evidence across all models indicated that Model 2 outperformed all other competing models, with a posterior probability exceeding 99%. Model 2 exhibited inherent connections involving both cortico-cerebellar coupling and subcortico-cerebellar coupling.

The cerebellum was added to the fully connected cortico-basal ganglia network model, and four models were created. The four models were subjected to fixed and random effects Bayesian model calculations. Model 2 eventually was shown to be the best because it had the most relevant logarithmic evidence. This also meant that the best model's posterior probability was greater than 99% in comparison to its nearest competitor (Figure 4). Model 2 included cortico-cerebellar and subcortical-cerebellar couplings, showing that the probability of a fully connected cortico-basal ganglia-cerebellum network model in PD patients in the resting state was greater than 99%.

### 3.4 Coupling strengths are correlated with clinical efficacy

All edges in the cortico-basal ganglia network had no correlation with the baseline UPDRS-III. The coupling strength of THA-M1 (R = 0.442, *P* = 0.009) and M1-STN (R = 0.481, *P* = 0.004) is associated with the improvement resulting from DBS. Detailed results are presented in Table 2.

**Table 2:** The relationship of coupling strength and clinical response.

**Items**	**Baseline**		**Improvement of DBS**	
	**UPDRS-III (OFF) (r)**	***P***	**Improved UPDRS-III (r)**	***P***
THA_M1	0.088	0.622	**0.442**	**0.009**
CEL_M1	0.205	0.245	0.280	0.109
M1_PUT	0.294	0.089	0.138	0.435
CEL_PUT	0.030	0.868	−0.165	0.350
PUT_THA	−0.058	0.746	−0.040	0.820
STN_THA	0.077	0.665	0.043	0.809
M1_STN	0.201	0.255	**0.481**	**0.004**
PUT_STN	0.151	0.393	−0.048	0.790
M1_CEL	0.138	0.437	0.208	0.238
THA_CEL	0.103	0.563	0.101	0.569

All values displayed with Pearson correlation coefficient (r). Positive correlations indicate that greater motor benefit correlates with higher coupling strength, as an improvement in UPDRS-III and the subscores are positive changes. *P* < 0.05, after FDR correction for multiple comparisons. The items marked in bold represent those with statistical significance.

The coupling strength of THA-M1 (R = 0.507, *P* = 0.002) and M1-STN (R = 0.461, *P* = 0.006) is associated with the improvement in rigidity due to DBS. Additionally, the coupling strength of PUT_THA (R = 0.387, *P* = 0.024), STN_THA (R = 0.439, *P* = 0.009), and CEL_THA (R = 0.395, *P* = 0.021) is related to the improvement in tremor from DBS. Furthermore, the coupling strength of M1-STN (R = 0.357, *P* = 0.038) is associated with the improvement in bradykinesia due to DBS. Specific details are available in Supplementary Table 3. The cerebellum-putamen pathway with baseline rigidity (*P* = 0.075) and the thalamus-M1 pathway with bradykinesia improvement (*P* = 0.062) also showed some correlation trends.

### 3.5 Coupling strengths predict clinical efficacy

**Figure 5:**
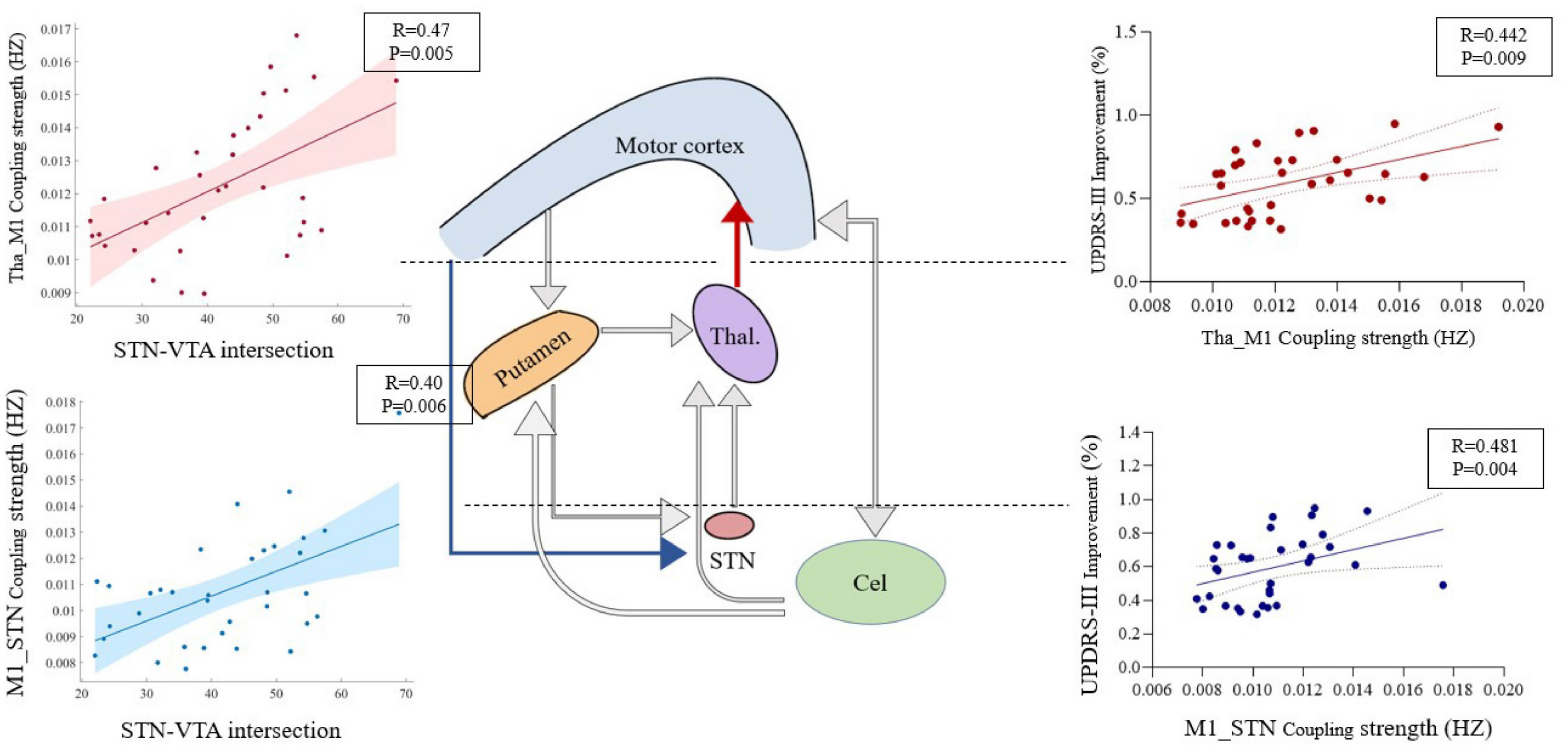
Association between network edges and UPDRS-III improvement. Red and blue arrows highlight the connections associated with clinical variables and STN-VTA intersections. Gray arrows represent connections from the connectivity matrix that were not found to be linked to clinical scores or STN-VTA intersections. In the graphs, the red data points and upward-trending lines depict the relationship between UPDRS-III improvement and the coupling strength from the thalamus to M1. Meanwhile, the blue data points and downward-trending lines illustrate the relationship between UPDRS-III improvement and the coupling strength from M1 to STN. Statistically significant relationships are denoted by *P* < 0.05 (FDR corrected).

A linear regression analysis showed that both the M1-STN pathway [overall model: R^2^ = 0.14, F = 5.112, *P* = 0.030, root mean square error (RMSE) = 14.9), and thalamus-M1 pathway (overall model: R^2^ = 0.23, F = 6.514, *P* = 0.018, and RMSE = 16.0] predicted changes in UPDRS-III (Figure 5). A multiple linear regression model was constructed to predict the DBS improvement in UPDRS-III using the coupling strength of THA-M1 and THA-STN. After leave-one-out cross-validation, the model showed a significant correlation between predicted and actual values (*p* = 0.003), with a cross-validated R^2^ of 0.18 and an RMSE of 15.2.

## 4 Discussion

The main findings of this paper were as follows: (1) individual STN-VTA intersections were important to DBS-induced movement enhancement; (2) the coupling strength of the Thal-M1 and M1-STN pathways in effective connectivities were related to the STN-VTA intersections; and (3) The preoperative individual-level coupling strength of Thal-M1 and M1-STN pathways was found to be associated with clinical improvements in PD patients. Additionally, the effective connection strength within the STN-thalamus-M1 pathway was able to predict clinical efficacy of DBS.

### 4.1 The efficacy of STN-DBS is related to the individualized VTA of the patient

Previous research suggested that the overlap between VTA and STN was related to DBS clinical efficacy (An et al., 2023). Our study confirmed the positive relationship between clinical improvement and the STN-VTA intersection. Notably, when the overlap was less than 20 mm^3^, the correlation between the therapeutic effect and VTA disappeared, indicating that STN-DBS may have had the lowest effective stimulation volume to have a clinical benefit. Therefore, to improve clinical outcomes, the target and stimulated VTA ranges should be aligned as much as possible with the STN.

### 4.2 Cortical-basal ganglia network construction

Using the DCM algorithm, we constructed a cortico-basal ganglia network model (Figure 4). It is generally accepted that the cerebellum and cortex gradually atrophy in PD patients (Filippi et al., 2020), and bradykinesia-related bidirectional projections exist between the cerebellum and M1 (Shen et al., 2020; Hanssen et al., 2019; Rajamani et al., 2024). Furthermore, the cerebellum has both anatomically and functionally connected projections to the basal ganglia, and participates in their outputs. The striatum and cerebellum have a causal degenerative structural connection that correlates with global movement, and the thalamus and cerebellum have a network of rigidly associated functional connections (Li et al., 2022; Quartarone et al., 2020; Sarasso et al., 2024). The findings of this study are consistent with previous results. The study expanded on the previous DCM model of the basal ganglia and provided a more comprehensive explanation of PD patients' effective connection state in the “resting” state.

The cortical-basal ganglia network constructed in this study had no significant correlation with disease severity, but it was significantly related to postoperative efficacy, suggesting the individual-specific network node defined by connectivity with the VTA was more responsive to DBS stimulation. Previous research found that functional connectivity generated in the canonical connectome dataset correlated with DBS motor outcomes when the VTA was used to characterize postoperative stimulation locations, implies that network characteristics defined by the VTA may not directly reflect individual differences in disease severities (Horn et al., 2017; Patrick et al., 2024). The results of the present study, however, differed from those of previous studies. While earlier studies suggested that neither group-level healthy nor Parkinsonian connectomes could fully capture the network engagement of a patient's individual stimulation site (Horn et al., 2017), our study utilized individualized fMRI data. We found that the individualized coupling strength of the M1-thalamus-STN pathway, defined by its connectivity with the patient-specific VTA, may combine with more established predictors such as levodopa responsiveness as a biomarker of STN-DBS responsiveness.

### 4.3 The M1-thalamus-STN pathway is associated with patient outcomes after DBS surgery

The hyperdirect pathway (M1-STN) is a critical monosynaptic cortical-subthalamic circuit involved in motor inhibition, as supported by prior work (Chen et al., 2020). Our study investigated the relationship between the hyperdirect pathway's effective connectivity strength and the overlap of VTA and STN. It has been reported that the greater the effective hyperdirect pathway's effective connection strength at the individual level, the better the postoperative efficacy of DBS, especially with rigidity. This is consistent with previous findings. Biophysical model studies have shown that there was a delay in the evoked response of the hyperdirect pathway in PD patients (Chen et al., 2020). Exaggerated high beta hyperdirect pathway activity can provoke the generation of widespread pathological synchrony at lower beta frequencies, which are implicated in the genesis of motor symptoms in PD (Oswal et al., 2021). Previous electrophysiological evidence has shown that STN-DBS could activate the monosynaptic connections from the cortex to the STN in PD patients, resulting in therapeutic effects. The closer the stimulus contacts are to the dorsolateral STN, the stronger the cortical evoked potentials in the primary motor cortex (Peeters et al., 2023, 2024). The intensity of the hyperdirect pathway in the initial state of PD patients can predict postoperative efficacy. This could be due to more precise STN stimulations, which can activate more monosynaptic connections mediated by the hyperdirect pathway, to improve the hyperdirect pathway's evoked delay and thus increase the hyperdirect pathway's effective connection strength. Our study therefore provided imaging evidence for the relationships between hyperdirect pathways and postoperative outcomes after DBS.

Furthermore, M1-STN was found to be positively associated with bradykinesia, but this finding did not explain the multiple comparison results. Bradykinesia is caused by a disruption in the functional connectivity of the hyperdirect pathway in PD patients (Jia et al., 2018). However, STN-DBS can increase the excitatory signal of the hyperdirect pathway, thereby improving bradykinesia (Sanders, 2017). Our findings were consistent with previous research, and involved a trend of effective VTA-M1 coupling strength to improve postoperative bradykinesia.

The thalamo-cortical pathway is the final output pathway affected in PD patients. The effective connection strength of the thalamus to the cortex was found to be positively correlated with the activation volume of the individualized STN, in our study. Furthermore, it was discovered that the effective thalamus-M1 connection was generated through the mediation of individualized STNs, the strength of which could predict the postoperative efficacies of DBS in patients, particularly improvements of rigidity. This is consistent with previous research results. M1 LFP recordings in PD patients have revealed increased beta band oscillations during the stop-phase of movement (Li et al., 2012; Crowell et al., 2012), as well as bursts of neural activity with long periods of silence in M1 LFP recordings. This widespread synchronization has been linked to changes in neuronal coupling strengths in the thalamo-cortical and other cortical motor networks (Weiss et al., 2015; Coutant et al., 2022). STN-DBS stimulation activates the cortico-thalamic pathway by increasing the thalamic reticular region to relay cell protruding coupling strength and inhibits beta band oscillations in the primary motor cortex (Farokhniaee and Lowery, 2021). According to our findings, the relationship between thalamus-M1 effective connectivity strength and postoperative efficacy may be related to the degree of synaptic damage to this pathway. Furthermore, the pathway's effective connection can reflect the strength of its synaptic-mediated pathway coupling from the image level.

While the VTA is a postoperative construct, our ‘post hoc' analysis employing it served a crucial mechanistic purpose: to achieve a more precise mapping of clinical improvement onto the specific neural circuitry activated by stimulation. This approach allowed us to identify that engagement of a network node defined by the intersection of the STN with the VTA is associated with motor benefit. Importantly, the efficacy of stimulating this node was correlated with—and could be anticipated by—the preoperative effective connectivity strength of the broader STN-Thal-M1 pathway. This key finding transitions the focus from a purely anatomical landmark (the STN) to a quantifiable circuit trait (STN-Thal-M1 connectivity) that is derivable from standard preoperative imaging. Consequently, our work lays the foundation for developing genuinely preoperative predictive models. Such models would utilize individual connectomic profiles to estimate the likelihood of clinical benefit from stimulating a given STN subregion, thereby advancing the paradigm toward personalized, connectome-informed DBS targeting.

### 4.4 The cerebellum is involved in postoperative tremor improvement

Some network edges, such as the cerebellum-thalamus, caudate-thalamus, and STN-thalamus, were associated with tremors, despite the fact that they could not be corrected for multiple comparisons. Previous research has reported that activations of the thalamus and cerebellum correlated with decreased tremors in patients, possibly as a result of the dentato-rubro-thalamic tract (DRT) action (Coenen et al., 2014). The DRT is a fiber bundle that connects the cerebellum to the basal ganglia (Lipp et al., 2022). It originates in the cerebellum and connects to the thalamus (Mollink et al., 2016), and it is essential for tremor control. Previous studies have reported that stimulating the ventral intermediate nucleus of the thalamus could effectively reduce functional coupling between the cerebellum and thalamus mediated by DRT, resulting in tremor control (Petersen et al., 2018; Akram et al., 2018; Gravbrot et al., 2020). In the present study, we characterized the cerebellum-thalamus and basal ganglia-thalamus connections. There was a clear trend of improvements in edges of the network and tremor, which may have resulted from activation of a portion of the DRT fiber bundles for individualized VTA stimulations. However, the stimulation range of the VTA was smaller than the overall STN nucleus and failed to stimulate the core portion of DRTT fiber bundles.

Although we included the cerebellum in our whole network model, there was no significant positive result for the cerebellum other than its presence in tremor improvement. This may be due to the fact that the cerebellum is more involved in functions such as balance and movement initiation in PD patients (Oswal et al., 2021). Because there was no clear indicator for these functions in the content of our evaluation, the cerebellum's role in the network is unclear.

### 4.5 Limitations

Our research had some limitations. We studied a small number of patients, which was limited by the need for patients to undergo MRI scans in a preoperative state without medication. We excluded patients who were unable to be scanned without medication, as well as patients with excessive head movements. Furthermore, we only measured the improvements of motor symptoms in patients, and did not measure other symptoms, such as the effects of DBS on cognition and emotion.

## 5 Conclusion

Our study showed that in PD patients, the degree of individual overlap between VTA and STN is related to DBS-induced clinical movement improvements, with a specific basis for functional connections. Moreover, M1-Thal-STN pathway effective connectivities at individual levels were predictive of movement improvements in PD patients, especially in their rigidities. Our results identified a predictive value of individual M1-Thal-STN pathway effective connectivities, and they provide a theoretical foundation for the development of individualized DBS plans and surgical optimizations.

## Data Availability

The raw data supporting the conclusions of this article will be made available by the authors, without undue reservation.
