# Supplementary material for: Individualized cortico-basal ganglia network effective connectivity predicts outcomes of STN-DBS in patients with Parkinson's disease
Source: Front Neurosci. 2026 Jan 14;19:1745334. doi: 10.3389/fnins.2025.1745334 (PMC12847302; doi:10.3389/fnins.2025.1745334)
Supplement: Supplementary file 1 [file Table_1.docx]

Supplementary Table1

|  | Baseline | | | | Follow-up | | | |
| --- | --- | --- | --- | --- | --- | --- | --- | --- |
|  | MDS-UPDRS-Ⅲ (med off) | Rigidity | Tremor | Bradykinesia | MDS-UPDRS-Ⅲ (med off) | Rigidity | Tremor | Bradykinesia |
| Sub_01 | 68 | 9 | 22 | 24 | 28 | 4 | 12 | 11 |
| Sub_02 | 63 | 10 | 16 | 16 | 52 | 7 | 13 | 20 |
| Sub_03 | 39 | 12 | 6 | 8 | 2 | 0 | 1 | 1 |
| Sub_04 | 79 | 12 | 18 | 29 | 63 | 7 | 12 | 26 |
| Sub_05 | 52 | 9 | 11 | 18 | 26 | 0 | 0 | 0 |
| Sub_06 | 64 | 9 | 23 | 23 | 27 | 5 | 9 | 11 |
| Sub_07 | 48 | 10 | 7 | 21 | 10 | 2 | 0 | 5 |
| Sub_08 | 91 | 15 | 6 | 44 | 51 | 12 | 3 | 21 |
| Sub_09 | 40 | 8 | 2 | 20 | 36 | 4 | 11 | 13 |
| Sub_10 | 29 | 4 | 0 | 14 | 29 | 4 | 0 | 14 |
| Sub_11 | 46 | 12 | 6 | 14 | 16 | 0 | 0 | 0 |
| Sub_12 | 90 | 13 | 25 | 28 | 24 | 1 | 9 | 9 |
| Sub_13 | 32 | 10 | 0 | 8 | 11 | 0 | 0 | 0 |
| Sub_14 | 86 | 13 | 25 | 33 | 8 | 2 | 1 | 2 |
| Sub_15 | 41 | 4 | 8 | 11 | 38 | 4 | 12 | 15 |
| Sub_16 | 41 | 8 | 2 | 14 | 28 | 5 | 0 | 9 |
| Sub_17 | 17 | 1 | 3 | 3 | 19 | 5 | 0 | 5 |
| Sub_18 | 72 | 10 | 19 | 25 | 5 | 0 | 0 | 4 |
| Sub_19 | 37 | 10 | 2 | 17 | 10 | 1 | 0 | 6 |
| Sub_20 | 33 | 5 | 9 | 15 | 19 | 5 | 2 | 10 |
| Sub_21 | 32 | 8 | 5 | 11 | 11 | 2 | 0 | 4 |
| Sub_22 | 36 | 9 | 2 | 19 | 6 | 0 | 0 | 5 |
| Sub_23 | 36 | 4 | 3 | 13 | 14 | 0 | 0 | 9 |
| Sub_24 | 66 | 11 | 12 | 31 | 39 | 7 | 8 | 16 |
| Sub_25 | 23 | 2 | 5 | 12 | 15 | 2 | 7 | 2 |
| Sub_26 | 57 | 6 | 22 | 20 | 42 | 5 | 14 | 18 |
| Sub_27 | 51 | 8 | 14 | 19 | 49 | 3 | 10 | 21 |
| Sub_28 | 30 | 4 | 4 | 12 | 19 | 3 | 0 | 7 |
| Sub_29 | 24 | 3 | 0 | 13 | 19 | 4 | 1 | 9 |
| Sub_30 | 38 | 9 | 1 | 18 | 24 | 4 | 0 | 14 |
| Sub_31 | 48 | 9 | 7 | 18 | 5 | 0 | 2 | 1 |
| Sub_32 | 87 | 16 | 5 | 44 | 26 | 2 | 2 | 12 |
| Sub_33 | 53 | 0 | 13 | 19 | 27 | 0 | 9 | 2 |
| Sub_34 | 89 | 20 | 3 | 43 | 33 | 5 | 0 | 19 |
| Sub_35 | 33 | 7 | 0 | 15 | 9 | 0 | 0 | 6 |
| Sub_36 | 74 | 11 | 20 | 30 | 26 | 4 | 6 | 9 |
| Sub_37 | 30 | 3 | 4 | 15 | 20 | 5 | 0 | 14 |
| Sub_38 | 59 | 9 | 2 | 32 | 38 | 10 | 0 | 22 |
| Sub_39 | 50 | 7 | 13 | 20 | 27 | 4 | 5 | 13 |
| Sub_40 | 39 | 8 | 3 | 16 | 11 | 0 | 0 | 1 |
| Sub_41 | 51 | 6 | 2 | 32 | 33 | 8 | 3 | 19 |
| Sub_42 | 54 | 7 | 19 | 19 | 19 | 0 | 0 | 2 |
| Sub_43 | 49 | 6 | 15 | 24 | 31 | 2 | 11 | 17 |

Supplementary Table2

|  | Left simulator clinical settings | | | | | Right simulator clinical settings | | | | |  |
| --- | --- | --- | --- | --- | --- | --- | --- | --- | --- | --- | --- |
|  | Amplitude  (V) | Frequency  (Hz) | Pulse Width(μs) | +Active contact | -Active contact | Amplitude  (V) | Frequency  (Hz) | Pulse Width  (μs) | +Active contact | -Active contact | manufacturer |
| Sub_01 | 2.00 | 90 | 60 | 4+ | 2- | 2.00 | 90 | 60 | 8+ | 6- | Pins |
| Sub_02 | 1.60 | 130 | 60 | 1+ | 3- | 1.70 | 130 | 60 | 5+ | 7- | Pins |
| Sub_03 | 1.50 | 130 | 60 | C+ | 3- | 1.65 | 130 | 60 | C+ | 10- | Medtronic |
| Sub_04 | 2.00 | 60 | 60 | C+ | 2- | 2.00 | 60 | 60 | C+ | 8- | Pins |
| Sub_05 | 1.80 | 60 | 60 | C+ | 3- | 1.80 | 60 | 60 | C+ | 7- | Pins |
| Sub_06 | 2.00 | 130 | 60 | C+ | 2- | 2.00 | 130 | 60 | C+ | 9- | Medtronic |
| Sub_07 | 1.90 | 130 | 60 | C+ | 3- | 2.05 | 130 | 60 | C+ | 7- | Pins |
| Sub_08 | 1.90 | 135 | 60 | C+ | 3- | 1.90 | 135 | 60 | C+ | 7- | Pins |
| Sub_09 | 1.70 | 125 | 60 | C+ | 3- | 1.70 | 125 | 60 | C+ | 7- | Pins |
| Sub_10 | 1.80 | 60 | 60 | C+ | 3- | 1.80 | 60 | 60 | C+ | 7- | Pins |
| Sub_11 | 1.80 | 60 | 60 | C+ | 3- | 1.80 | 60 | 60 | C+ | 7- | Pins |
| Sub_12 | 2.00 | 130 | 60 | C+ | 2- | 2.00 | 130 | 60 | C+ | 9- | Medtronic |
| Sub_13 | 1.80 | 60 | 60 | C+ | 3- | 1.80 | 60 | 60 | C+ | 7- | Pins |
| Sub_14 | 1.60 | 120 | 60 | C+ | 2- | 1.80 | 120 | 60 | C+ | 6- | Pins |
| Sub_15 | 1.60 | 130 | 60 | C+ | 4- | 1.60 | 130 | 60 | C+ | 8- | Medtronic |
| Sub_16 | 1.80 | 130 | 60 | C+ | 1- | 1.80 | 130 | 60 | C+ | 9- | Medtronic |
| Sub_17 | 1.60 | 130 | 60 | 3+ | 1- | 1.60 | 130 | 60 | 11+ | 9- | Medtronic |
| Sub_18 | 1.70 | 130 | 60 | C+ | 3- | 1.60 | 130 | 60 | C+ | 8- | Medtronic |
| Sub_19 | 1.70 | 130 | 60 | C+ | 3- | 1.70 | 130 | 60 | C+ | 7- | Pins |
| Sub_20 | 1.40 | 130 | 60 | C+ | 1- | 1.40 | 130 | 60 | C+ | 9- | Medtronic |
| Sub_21 | 1.65 | 130 | 60 | C+ | 2- | 1.65 | 130 | 60 | C+ | 6- | Pins |
| Sub_22 | 1.70 | 132 | 60 | C+ | 3- | 1.60 | 132 | 60 | C+ | 8- | Pins |
| Sub_23 | 1.80 | 130 | 60 | C+ | 2- | 1.80 | 130 | 60 | C+ | 10- | Medtronic |
| Sub_24 | 1.50 | 130 | 60 | C+ | 3- | 1.80 | 130 | 60 | C+ | 6- | Pins |
| Sub_25 | 1.50 | 130 | 60 | C+ | 3- | 1.50 | 130 | 60 | 8+ | 10- | Medtronic |
| Sub_26 | 1.60 | 130 | 60 | C+ | 2- | 1.60 | 130 | 60 | C+ | 10- | Medtronic |
| Sub_27 | 1.50 | 130 | 60 | C+ | 1- | 1.50 | 130 | 60 | C+ | 9- | Medtronic |
| Sub_28 | 0.70 | 130 | 60 | C+ | 2- | 1.50 | 130 | 60 | C+ | 6- | Pins |
| Sub_29 | 1.80 | 130 | 60 | C+ | 3- | 1.50 | 130 | 60 | C+ | 7- | Pins |
| Sub_30 | 1.80 | 130 | 60 | C+ | 2- | 1.80 | 130 | 60 | C+ | 10- | Medtronic |
| Sub_31 | 1.30 | 130 | 60 | C+ | 3- | 1.50 | 130 | 60 | C+ | 8- | Pins |
| Sub_32 | 1.80 | 130 | 60 | C+ | 3- | 1.80 | 130 | 60 | C+ | 7- | Pins |
| Sub_33 | 1.80 | 130 | 60 | C+ | 3- | 1.80 | 130 | 60 | C+ | 7- | Pins |
| Sub_34 | 2.00 | 130 | 80 | C+ | 3- | 2.10 | 130 | 80 | C+ | 6- | Pins |
| Sub_35 | 3.05 | 145 | 90 | C+ | 1- | 1.8 | 145 | 60 | C+ | 9- | Medtronic |
| Sub_36 | 2 | 130 | 60 | C+ | 3- | 2 | 130 | 60 | C+ | 7- | Pins |
| Sub_37 | 2 | 130 | 60 | C+ | 1- | 2 | 130 | 60 | C+ | 9- | Medtronic |
| Sub_38 | 1.6 | 130 | 60 | C+ | 3- | 2 | 130 | 60 | C+ | 7- | Pins |
| Sub_39 | 2.15 | 140 | 70 | C+ | 2-3- | 1.8 | 140 | 70 | C+ | 7- | Pins |
| Sub_40 | 2 | 130 | 60 | C+ | 3- | 2 | 130 | 60 | C+ | 7- | Pins |
| Sub_41 | 1 | 130 | 60 | C+ | 3- | 1.15 | 130 | 60 | C+ | 7- | Pins |
| Sub_42 | 2 | 130 | 60 | C+ | 3- | 2 | 130 | 60 | C+ | 7- | Pins |
| Sub_43 | 3 | 130 | 60 | C+ | 4- | 3 | 130 | 60 | C+ | 8- | Pins |

Supplementary Table3

|  | Baseline | | | | | | | improvement of DBS | | | | |  |
| --- | --- | --- | --- | --- | --- | --- | --- | --- | --- | --- | --- | --- | --- |
|  | rigidity | P | tremor | P | bradykinesia | | P | rigidity | P | tremor | P | bradykinesia | P |
| THA_M1 | 0.087 | 0.625 | 0.122 | 0.491 | -0.16 | 0.366 | | **0.507^a^** | **0.002** | 0.187 | 0.29 | 0.324 | 0.062 |
| CEL_M1 | 0.129 | 0.466 | 0.135 | 0.445 | -0.015 | 0.932 | | 0.155 | 0.38 | -0.027 | 0.879 | 0.203 | 0.251 |
| M1_PUT | 0.044 | 0.803 | 0.265 | 0.129 | 0.175 | 0.323 | | 0.162 | 0.361 | 0.013 | 0.941 | 0.167 | 0.345 |
| CEL_PUT | -0.309 | 0.075 | -0.023 | 0.896 | 0.053 | 0.767 | | -0.155 | 0.382 | 0.044 | 0.804 | -0.05 | 0.779 |
| PUT_THA | -0.017 | 0.922 | 0.042 | 0.815 | -0.14 | 0.429 | | 0.071 | 0.69 | **0.387** | **0.024** | 0.008 | 0.964 |
| STN_THA | 0.107 | 0.545 | 0.031 | 0.863 | -0.039 | 0.825 | | 0.226 | 0.198 | **0.439** | **0.009** | 0.154 | 0.385 |
| M1_STN | 0.243 | 0.166 | -0.029 | 0.872 | 0.069 | 0.699 | | **0.461^a^** | **0.006** | 0.254 | 0.147 | **0.357** | **0.038** |
| PUT_STN | 0.032 | 0.856 | 0.001 | 0.996 | 0.237 | 0.178 | | -0.028 | 0.874 | 0.207 | 0.24 | 0.066 | 0.711 |
| M1_CEL | 0.143 | 0.42 | 0.052 | 0.77 | -0.035 | 0.846 | | 0.084 | 0.639 | 0.065 | 0.715 | 0.036 | 0.838 |
| CEL_THA | 0.168 | 0.341 | -0.074 | 0.679 | 0.021 | 0.908 | | 0.144 | 0.417 | **0.395** | **0.021** | 0.175 | 0.321 |

All values displayed with Pearson correlation coefficient (r). Positive correlations indicate that greater motor benefit correlates with higher coupling strength, as an improvement in UPDRS-III and the subscores are positive changes.

**^a^** represents the P value after FDR correction. uncorrected *P* < 0.05.
